# Skin Barrier Function and *Staphylococcus aureus* Colonization in Vestibulum Nasi and Fauces in Healthy Infants and Infants with Eczema: A Population-Based Cohort Study

**DOI:** 10.1371/journal.pone.0130145

**Published:** 2015-06-12

**Authors:** Teresa Løvold Berents, Karin Cecilie Lødrup Carlsen, Petter Mowinckel, Håvard Ove Skjerven, Bente Kvenshagen, Leif Bjarte Rolfsjord, Maria Bradley, Agne Lieden, Kai-Håkon Carlsen, Peter Gaustad, Petter Gjersvik

**Affiliations:** 1 Institute of Clinical Medicine, University of Oslo, Oslo, Norway; 2 Department of Dermatology, Oslo University Hospital, Oslo, Norway; 3 Department of Pediatrics, Oslo University Hospital, Oslo, Norway; 4 Department of Pediatrics, Østfold Hospital, Fredrikstad, Norway; 5 Department of Pediatrics, Innlandet Hospital Trust, Elverum, Norway; 6 Department of Molecular Medicine, Karolinska Institute at Karolinska University Hospital, Stockholm, Sweden; 7 Department of Dermatology, Karolinska University Hospital, Stockholm, Sweden; 8 Department of Microbiology, Oslo University Hospital, Oslo, Norway; University Hospital Hamburg-Eppendorf, GERMANY

## Abstract

Atopic eczema (AE) is associated with *Staphylococcus aureus* (*S*. *aureus*) colonization and skin barrier dysfunction, often measured by increased transepidermal water loss (TEWL). In the present study, the primary aim was to see whether *S*. *aureus* colonization in the vestibulum nasi and/or fauces was associated with increased TEWL in infants with healthy skin and infants with eczema. Secondarily, we aimed to investigate whether TEWL measurements on non-lesional skin on the lateral upper arm is equivalent to volar forearm in infants. In 167 of 240 infants, recruited from the general population, TEWL measurements on the lateral upper arm and volar forearm, using a DermaLab USB, fulfilled our environmental requirements. The mean of three TEWL measurements from each site was used for analysis. The infants were diagnosed with no eczema (n = 110), possible AE (n = 28) or AE (n = 29). DNA samples were analysed for mutations in the *filaggrin* gene (*FLG)*. Bacterial cultures were reported positive with the identification of at least one culture with *S*. *aureus* from vestibulum nasi and/or fauces. *S*. *aureus* colonization, found in 89 infants (53%), was not associated with increased TEWL (i.e. TEWL in the upper quartile), neither on the lateral upper arm or volar forearm (p = 0.08 and p = 0.98, respectively), nor with AE (p = 0.10) or *FLG* mutation (p = 0.17). TEWL was significantly higher on both measuring sites in infants with AE compared to infants with possible AE and no eczema. *FLG* mutation was significantly associated with increased TEWL, with a 47% difference in TEWL. We conclude that *S*. *aureus* in vestibulum nasi and/or fauces was not associated with TEWL, whereas TEWL measurements on the lateral upper arm and volar forearm appear equally appropriate in infants.

## Introduction

Atopic eczema (AE) is a common skin disease and is characterized by dry skin, pruritus, and eczematous skin [1,2]. In infants with AE, the eczema is frequently located in the face and/or scalp. In older children, the eczema more often has a flexural distribution, although lateral part of the upper arms may also be involved [3–7]. The onset of AE is usually early in life, with a majority being reported before the age of 12 months [1].

The pathogenesis of AE is complex, involving skin barrier dysfunction, immune dysregulation, cutaneous inflammation and colonization with *Staphylococcus aureus* (*S*. *aureus)* [1,8–10]. Dysfunction of the skin barrier leads to increased water loss and increased entry of allergens, toxins, and infectious microorganisms [1]. Skin barrier function can be evaluated by measuring transepidermal water loss (TEWL) [11]. Increased TEWL, indicating a skin barrier dysfunction, is often found in patients with AE [12,13], but has also been reported in infants with dry skin and mutation in the gene encoding for the profilaggrin protein [14]. Filaggrin deficiency is a major predisposing factor for AE [15] and may also be an independent increased risk factor for *S*. *aureus* colonization [16].

Increased TEWL has been found to be associated with *S*. *aureus* colonization on clinically normal skin in adults [17]. In infants, the *S*. *aureus* colonization rate in healthy skin is low [18]. *S*. *aureus* is thought to act as a superantigen contributing to the development of AE [19]. Patients with AE, regardless of age, are frequently colonized by *S*. *aureus* [1,19–24], and the number of colonization sites, has been associated with the severity of AE [12,23,25] The vestibulum nasi could be a reservoir of *S*. *aureus*, with the bacteria spreading to the skin by autotransmission [20,25–27]. Also, nasal colonization of *S*. *aureus* at six months of age has been reported to be associated with an increased risk of AE in the 2nd year of life [28]. In a study on patients and staff at an orthopedic ward in Sweden, *S*. *aureus* colonization was more frequent in the throat than in anterior nares [29].

TEWL may vary by anatomical site [11,30–32]. TEWL is most often measured on the volar forearm [30]. In infants, however, AE in infancy is often located on the lateral part of upper arm [6,7], suggesting that the skin barrier may better reflect possible disease association at this loctation rather than at the volar forearm that is more often free of eczema manifestation in infants. Standard temperature and humidity conditions as well as a calm baby are vital for measurements of TEWL. Thus, easy access to the lateral upper arm, reducing the risk of excess heat or humidity by the infant flexing the lower arm, and a location more often associated with atopic eczema manifestation renders the lateral upper arm as a suitable site for TEWL measurements in infants. We are not aware of studies comparing TEWL on volar forearm and TEWL on lateral upper arm in infants.

The main objective of the present study was to investigate if *S*. *aureus* colonization in vestibulum nasi and/or fauces, potentially preceding AE, is associated with skin barrier dysfunction as assessed by TEWL measurements in infants recruited from a general population. The secondary aims were to investigate if TEWL measurements on the lateral upper arm and volar forearm provide similar associations between TEWL and AE and to assess correlations between four common *FLG* mutations and *S*. *aureus* colonization and TEWL measurements.

## Materials and Methods

The present study included infants from a general population, serving as a control population of the Bronchiolitis All SE-Norway study [33], registered at ClincialTrials.gov number NCT00817466, EudraCT number, 2009-012667-34. A letter of invitation was sent to the parents of 2,400 infants, randomly selected from the general population register of Oslo and Fredrikstad, two cities in the south east of Norway. Of these, 240 responded and were enrolled. The inclusion criterion was age 12 months or younger at the time of recruitment. Exclusion criteria were any cardiac, pulmonary (other than obstructive airways disease), immunological, neurological or oncological disease assumed to affect an infant’s eczema.

The infants were examined within three months from March 2012. One or both parents were interviewed using a structured questionnaire that included questions on previous and current health and diseases of the child and family members, socio-economic status, quality of life and environmental exposures.

The study was approved by the Regional Committee for Medical and Health Research Ethics, the biobank was registered appropriately, and informed written consent was obtained from all caregivers.

### Subjects

Of the 240 enrolled infants, this study includes the 167 infants (mean age 6.4 months) whose TEWL measurements fulfilled our requirements for temperature and humidity (Table 1). Subjects were excluded due to crying (n = 7) or non-optimal measuring conditions, i.e. humidity (n = 66) and/or temperature (n = 35).

**Table 1:** Characteristics.

	Included infants n = 167	Excluded infants n = 73	p-values
**Male**	87 (52)	47 (64)	0.08
**Age (months; min-max)**	6.4 (1.1–13.4)	6.9 (1–13.6)	0.26
**No eczema**	110 (66)	49 (67)	0.63
**Possible atopic eczema**	28 (17)	9 (12)	0.63
**Atopic eczema**	29 (17)	15 (21)	0.63
**Mother Caucasian**	156 (93)	70 (96)	0.58
**Father Caucasian**	152 (92)	68 (93)	0.58
**Smoking at home**	5 (3)	5 (7)	0.18
**Cat at home**	18 (11)	10 (14)	0.56
**Dog at home**	14 (9)	11 (16)	0.11
**Parental eczema**	62 (37)	15 (21)	0.01
**Parental asthma**	45 (27)	20 (27)	0.94
**Parental rhinitis**	92 (55)	40 (55)	0.97
***Filaggrin* mutation**	14 (8)	2 (3)	0.11
***S*. *aureus* vestibulum nasi**	47 (28)	13 (18)	0.09
***S*. *aureus* fauces**	73 (44)	28 (38)	0.43
***S*. *aureus* vestibulum nasi/fauces**	89 (53)	33 (45)	0.25

Characteristics of 240 infants, recruited from the general population in Oslo and Fredrikstad, Norway, of whom 167 were included and 73 excluded from the analyses. Exclusions were done due to crying during the measuring transepidermal water loss (n = 7) and/or measuring conditions not fulfilling strict environmental criteria for humidity and/or temperature (n = 66). All values are given as number (percentage), unless otherwise stated.

Experienced and trained physicians performed the clinical examinations at one visit. All skin assessments were performed strictly by validated scoring criteria in collaboration with a dermatologist. AE was diagnosed according to the criteria of Hanifin & Rajka, requiring the presence of minimum three of four major criteria and minimum three of 23 minor criteria [5]. Infants with clinical eczema, but not meeting these criteria, were classified as having possible AE. The SCORing Atopic Dermatitis index (SCORAD index) was used to score disease severity in those with AE [34]. Reported SCORAD is the mean of the score by the primary investigator (physician) and the secondary investigator (physician or trained study nurse).

### TEWL meaurements

TEWL was measured on non-lesional skin on the lateral part of upper arm and volar forearm, using the open chamber DermaLab USB (Cortex, Hadsund, Denmark) system. The infants were first acclimatized to the examination room for at least 15 minutes, wearing a diaper only. Investigations were performed while attempting a room temperature of 20–22°C and humidity of 40% [30]. Using different examination rooms, it was not possible to maintain these air humidity and temperature requirements in a strict manner. We accepted measurements performed at temperature 20–25°C, in line with a study of Kelleher et al [35], and humidity from 20 to 50%. Measurements were disregarded if the infants were crying. TEWL values were reported as the mean of three measurements performed for each test site.

### Bacterial cultures

Samples for bacterial cultures, using ESwab (Copan, Brescia, Italia), were taken from both the vestibulum nasi and fauces and cultivated on a blood agar plate as well as a selective plate for *S*. *aureus* (mannitol-salt agar) for two days. Growth of *S*. *aureus* was identified by a rapid agglutination test (Pastorex Staph Plus, Alere, UK) and registered semi-quantitatively. Samples from vestibulum nasi and/or fauces in one subject were reported as positive with at least one culture with *S*. *aureus* being identified. In infants with AE, bacterial cultures were also taken from eczematous skin.

### Genotyping

Blood samples were drawn from all infants using standard procedure. DNA was isolated from peripheral whole blood using QIAsymphony DNA Midi KIT (QIAGEN Instruments AG, Switzerland). All samples were screened for the four most common *FLG* mutations in the European population, i.e. R501X, 2282del4, R2447X and S3247X, using TaqMan allelic discrimination assays with minor modifications [36,37]. Two DNA plates (for a maximum of 174 samples) were genotyped twice for each mutation. The genotyping concordance was 100% when comparing samples that received a genotype call in both runs.The genotyping success rate for R501X, 2282del4, R2447X, S3247X and combined null was 99.7, 99.3, 97.9, 96.0, and 93.4%, respectively.

### Statistical analysis

Data are presented as percentages, except for continuous data, which are presented as estimated means with 95% confidence intervals (CI), unless otherwise stated. As TEWL values were not normally distributed, comparisons between groups were performed by robust regression analyses [38]. Comparison of infants with no eczema, possible AE and AE with regard to *S*. *aureus* colonization was performed by Chi square tests or robust one-way ANOVA, as appropriate.

As the cohort was established to serve as a control cohort for another study [33], a post-hoc power analysis was performed for *S*. *aureus* colonization and TEWL. Comparing group sizes of 129, 34 and 35 of three groups with mean TEWL values of 7.9, 8.4 and 10.8 g/m^2^h^-1^ would achieve 82% power to detect significant differences using a standard deviation of 9.7.

Due to difficulties in obtaining required measurement conditions, post-hoc analyses of the potential impact of temperature and humidity on TEWL measurements were performed, using Spearman correlation coefficient. These analyses revealed weak associations with temperature (r = 0.16; p = 0.002), but not with humidity (r = 0.052; p = 0.48). We therefore repeated the analyses including all 198 infants with TEWL measurements performed under the chosen temperature criteria, with results given in the online supplements (S1 and S2 Tables).

The level of statistical significance was set to 0.05. Statistical analysis was performed using IBM Statistical Package for Social Sciences (IBM SPSS Statistics, Version 21.0.1. Armonk, NY: IBM Corp) version 21.0 and Number Cruncher Statistical System (NCSS Inc., Kaysville, Utah), version 9.09.

## Results

The 167 infants included in the analyses were similar to the 73 excluded infants with respect to demographic and clinical parameters, except for more frequent parental eczema among the included infants (Table 1). Of the 167 included infants, 110 (66%) had no eczema, 28 (17%) possible AE and 29 (17%) AE. In infants with AE, the median SCORAD was 16.5 (range 7.5, 39.0).

The mean TEWL levels were significantly higher in infants with AE than in those with possible AE and with no eczema on both measurement sites (lateral upper arm p<0.001 and volar forearm p = 0.02, respectively) (Table 2). In infants with no eczema, the mean TEWL was 7.7 g/m^2^h^-1^ (95% CI 7.1, 8.4) on the lateral upper arm and 9.3 (8.4,10.2) on the volar forearm (p<0.001), with no significant differences in TEWL between the two measuring sites in those with possible AE and AE.

**Table 2:** Transepidermal water loss (TEWL).

	No eczema n = 110	Possible AE n = 28	AE n = 29	Overall p-value
**Lateral upper arm**	7.7 (7.1,8.4)	8.5 (7.2,9.8)	11.0^a^ (9.7,12.4)	0.0002
**Volar forearm**	9.3 (8.4,10.2)	9.2 (7.4,10.9)	12.8^a^ (11.1,14.6)	0.02

Transepidermal water loss (TEWL) in 167 infants, recruited from the general population in Oslo and Fredrikstad, Norway, with no eczema, possible atopic eczema (AE) and AE. Measurements were performed three times and recorded as mean g/m^2^h^-1^ each site. Results are given as estimated mean (95% CI) from robust regression analysis.

^a^p<0.001 AE vs. possible AE. There was no significant difference between possible AE and no eczema.

*S*. *aureus* colonization was observed in 89 infants (53%), i.e. 73 infants (44%) with colonization in the fauces and 47 (28%) with colonization in vestibulum nasi; 31 infants (19%) were colonized in both fauces and vestibulum nasi. The *S*. *aureus* colonization rates in vestibulum nasi and fauces were non-significantly higher in infants with AE compared to infants with possible AE and no eczema (vestibulum nasi 41, 30, 25%, respectively; p = 0.20; and fauces 55, 48, 40%, respectively; p = 0.30). The *S*. *aureus* colonization rate in the vestibulum nasi and fauces combined was non-significantly higher in infants with AE (69%) compared to infants with possible AE (57%) and no eczema (48%) (p = 0.12). Of the 29 infants with AE, eight were colonized with *S*. *aureus* on eczematous skin; all of these were colonized in the fauces and five in vestibulum nasi.

**Fig 1:**
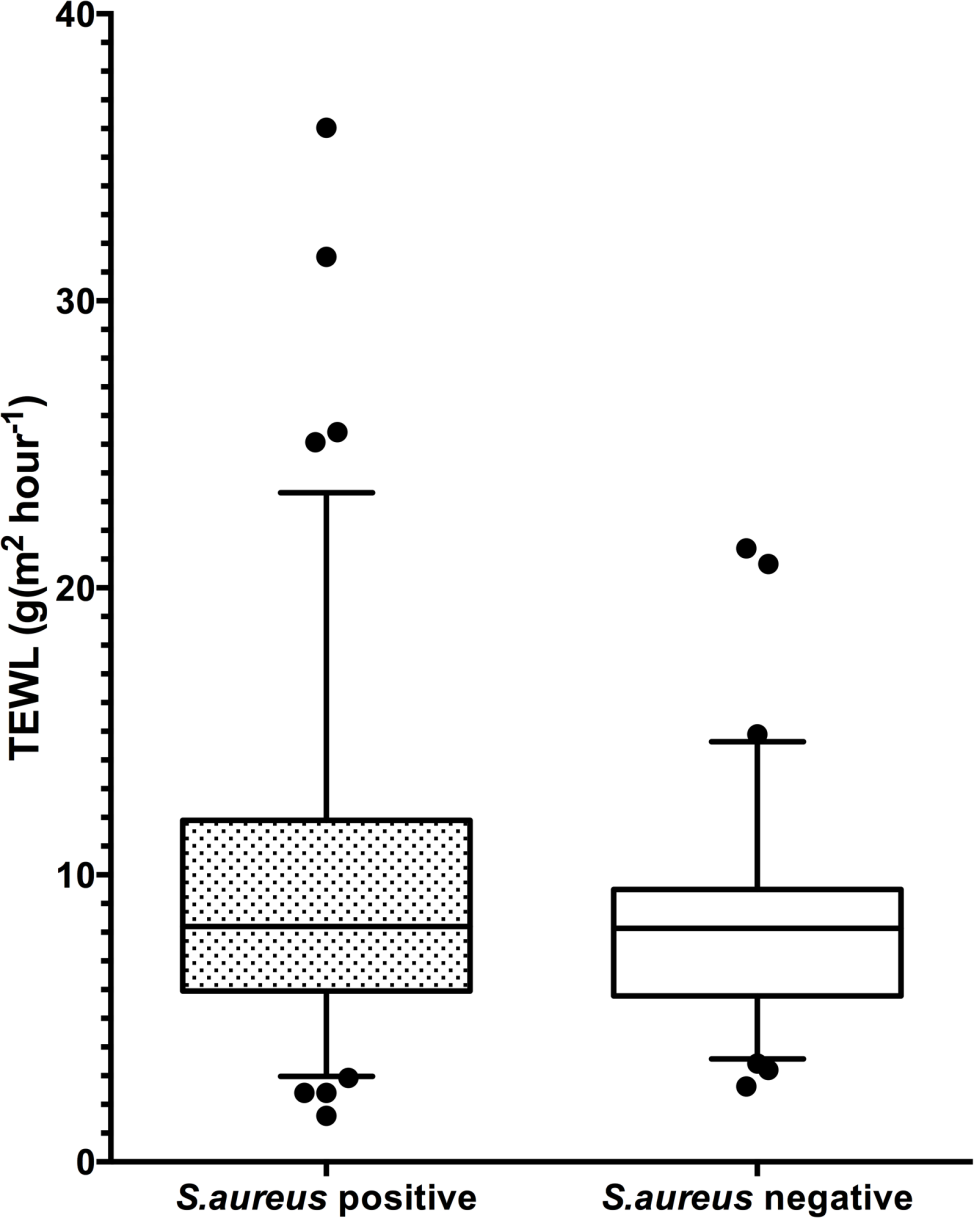
*Staphylococcus aureus* colonization and transepidermal waterloss. Boxplot showing *Staphylococcus aureus* (*S*. *aureus*) colonization in vestibulum nasi and/or fauces in 89 (53%) of 167 infants recruited from the general population was not significantly associated with the estimated mean transepidermal waterloss (TEWL; g/m^2^h^-1^) on the lateral upper arm.

There was no significant association between *S*. *aureus* colonization in vestibulum nasi and/or fauces and high TEWL (i.e. upper quartile) on lateral upper arm, as well as on volar forearm (p = 0.08 and p = 0.98, respectively), both among all infants (Fig 1) and after stratification for no eczema, possible AE and AE (all p>0.1). In infants with no eczema, the *S*. *aureus* colonization rate was 59% in infants with high TEWL (i.e. upper quartile) on the lateral upper arm and 48% in infants with low TEWL (i.e. lower quartile) (p = 0.41). In infants with AE, there was no significant association between *S*. *aureus* colonization rates in vestibulum nasi and/or fauces or eczematous skin and TEWL.

**Fig 2:**
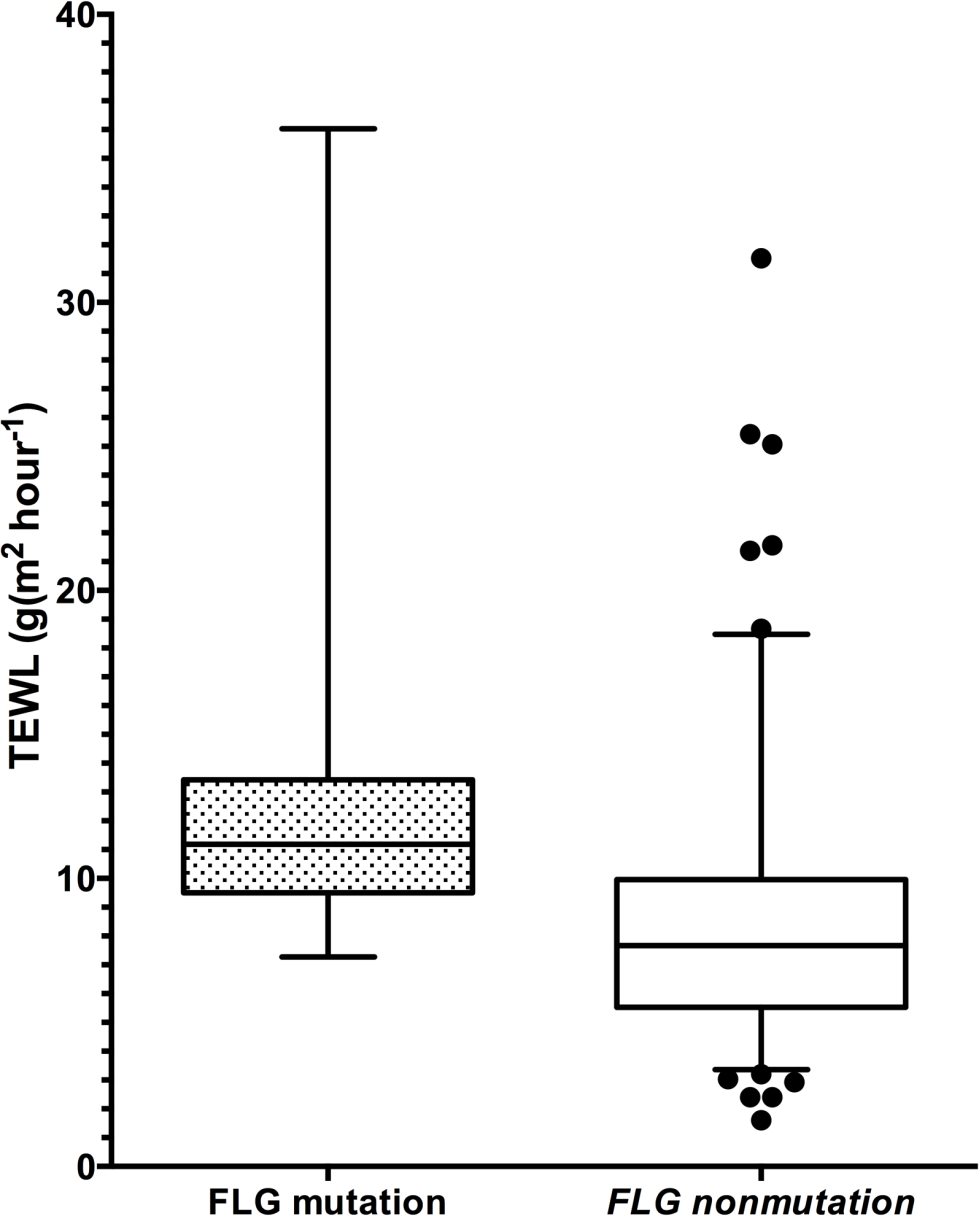
Transepidermal waterloss and *filaggrin* mutation. Boxplot showing *filaggrin* (*FLG*) mutation identified in 14 (8%) of 167 infants recruited from the general population and transepidermal water loss (TEWL; g/m^2^h^-1^) on the lateral upper arm. *FLG* mutations were associated with increased TEWL (p = 0.006).

Loss of function mutations in the *FLG* gene, i.e. R501X, 2282del4, R2447X and S3247X, identified in 14 infants (8%), all heterozygous, was associated with increased TEWL both on the lateral upper arm and volar forearm (p = 0.006 and p = 0.03, respectively) (Fig 2). The *FLG* mutation was not significantly associated with *S*. *aureus* colonization in any of the groups with respect to no eczema, possible or AE, or with disease severity in those with AE (all p>0.1).

Results regarding *S*. *aureus* colonization and TEWL remained largely unchanged when infants with TEWL measurements not fulfilling strict humidity requirements were included in the analysis, enlarging the cohort to 198 infants (S1 Text, S1 and S2 Tables). Also, when including all enrolled infants, resulting in a cohort of 240 infants, the association between *S*. *aureus* colonization and AE remained largely unchanged.

## Discussion

In the present study, *S*. *aureus* colonization in vestibulum nasi and/or fauces, observed in 53% of infants recruited from the general population, was not significantly associated with increased TEWL, nor with AE or *FLG* mutation. Measurements of TEWL appeared to be equally appropriate on the lateral upper arm as on the volar forearm.

The relatively high rate of *S*. *aureus* colonization in the vestibulum nasi and/or fauces in our cohort was in line with a Danish population based cohort study reporting a nasal colonization of 51.3% in 2–11 week age infants [39], but was higher than the nasal colonization of 21.1% described in a Dutch prospective birth cohort study at six months age [28]. In the present study, we combined the colonization rates of vestibulum nasi and fauces, as the fauces may be a major carriage site of *S*. *aureus* in addition to vestibulum nasi [29]. In the Danish study, infants were recruited among infants whose mothers had asthma, of whom about half also had AE [39]. The Dutch study was a prospective cohort study from fetal life until young adulthood, with a randomly selected cohort with a low prevalence of parental eczema [28]. Our cohort was recruited from the general population, although with some unintentional bias towards including more infants of parents with eczema. These differences may explain the different rates of *S*. *aureus* colonization in the three studies.

In the present study, eight infants with AE and *S*. *aureus* colonization on eczematous skin were also colonized in the vestibulum and/or fauces. Although the numbers are small, this is in line with studies where eczematous skin in patients with AE were colonized with the same bacterial strain as in the vestibulum nasi and/or fauces [25,26]. The nostrils is a source for *S*. *aureus* skin colonization [25,27]. These findings could be explained by autotransmission of *S*. *aureus*.

In the present study, *S*. *aureus* colonization rates in vestibulum nasi and/or fauces was non-significantly higher in infants with AE than in those with possible AE and no eczema. Several factors may contribute to *S*. *aureus* colonization in infants with AE, including immunological responses [40,41]. In AE, there is a Th2 response with increased levels of cytokines, contributing to the susceptibility to *S*. *aureus* in AE [41–43].

The lack of association between TEWL and *S*. *aureus* colonization in vestibulum nasi and/or fauces in infants with no eczema, possible AE and AE has to our knowledge not been reported previously. Others have reported an association between *S*. *aureus* colonization on the cheek and forehead and increased TEWL in healthy adults with normal skin [17]. Also, in a Swedish study of adult patients with AE, *S*. *aureus* skin colonization was significantly associated with TEWL on eczematous skin [12]. In Korean AE patients aged 1–40 years old, *S*. *aureus* colonization in anterior nares was reported to be non-significantly correlated with high TEWL [25]. The apparent discrepancies between these studies may have several explanations. First, age may modify the results, as adults generally have lower TEWL than infants [44]. Also, sampling sites and circumstances were different. In our study, *S*. *aureus* was detected in vestibulum nasi and fauces and TEWL measured on the lateral upper arm and volar forearm in infants with and without eczema, in contrast to the other studies, where *S*. *aureus* was detected on normal and eczematous skin, respectively. Although an association between *S*. *aureus* colonization in vestibulum nasi and/or fauces and TEWL in infants cannot be ruled out, the present study had a sufficient statistical power to detect significant differences of 82%. Our findings do not support a hypothesis that increased susceptibility to *S*. *aureus* in the vestibulum nasi and/or fauces is associated with increased TEWL and precedes the development of AE.

TEWL measurements on the lateral upper arm differentiated well between no, possible AE and AE, and appeared to be at least as good, or possibly better, than measurements performed at the standard site of the volar forearm. We are not aware of other studies using this site for TEWL measurements in infants. In line with other studies, TEWL was increased in infants with AE compared to infants with possible AE and no eczema for both measuring sites [13]. Having a *FLG* mutation was associated with high TEWL, but did not modify the results. These findings may have several implications. Cohort studies assessing pre-morbid skin barrier function for potential disease prediction would benefit from using standard sites that are commonly associated with development of AE, i.e. the lateral upper arm [6,7]. Also, the lateral upper arm is easily accessible during nursing of infants, improving the feasibility of measurements in a relaxed and content infant. Our study therefore indicates that TEWL measurements on lateral upper arm may be as appropriate for the evaluation of skin barrier function in infants as TEWL measurements on volar forearm.

TEWL measurements were performed using an open chamber system, enabling consecutive measurements [45]. The observed mean TEWL in our infants without eczema were slightly higher than, but generally in line with those observed in a cohort study with more than 1,000 new-born infants in Ireland, examined shortly after birth and using another open chamber system [35]. Open chamber devices require an environment without air turbulence, in contrast to the closed chamber systems, either an unventilated chamber or a condensed chamber system. Unventilated chamber systems compare ambient humidity with the humidity within the chamber after a filling time, but require some time to evaporate before starting the next measurement. Condensed chamber systems measure water vapor flux density and can make consecutive measurements. In vitro comparisons have shown a good correlation between various open chamber systems and condensed chamber systems with titrated water flux [45]. Open chamber systems appear more sensitive to changes in TEWL in the lower range [46]. Thus, all three measuring systems appear valid in research, but results should not be directly compared [45].

All infants were acclimatized to the ambient environment for at least 15 minutes, in line with present guidelines [30]. Even though great care was taken to achieve strict and stable conditions, it was not possible to meet the temperature and air humidity criteria for all infants, with the subsequent exclusion of many infants from the main analysis. The temperature was slightly higher than recommended in the guidelines, but similar to the temperature in the study by Kelleher et al [35]. The importance of air humidity in TEWL measurements has resulted in recommendations of relative air humidity to approximately 40% [30]. However, results from studies on the impact of humidity on TEWL are somewhat conflicting [47,48]. We therefore retrospectively evaluated the potential impact of humidity and temperature on TEWL measurements, finding that humidity was not significantly associated with the TEWL measurements, whereas temperature was slightly associated (on line supplement only). The largely unchanged results of our repeated analyses including infants whose TEWL measurements had been performed under strict temperature criteria (20–25°C, mean 23.7°C), but allowing for indoor relative humidity levels between 14 and 64% (mean 31.7%), may question the present recommendations regarding indoor humidity. In our opinion, these requirements should be balanced against the feasibility of measuring TEWL in infants.

## Conclusion

In the present study with infants recruited from a general population, *S*. *aureus* colonization in vestibulum nasi and/or fauces was not associated with TEWL measured on the arm, or with atopic eczema. Measuring TEWL on the lateral upper arm appears to be appropriate for the evaluation of skin barrier function in infants.

## Supporting Information

S1 Table Characteristics. Characteristics of 198 included and 42 excluded infants enrolled as a control population for the Bronchiolitis All SE-Norway study. Infants were excluded due to crying (n = 7) and/or not fulfilling strict environmental criteria for humidity and/or temperature (n = 35). All values are given as number (percentage), unless otherwise stated.
(DOCX)

S2 Table Transepidermal water loss (TEWL). Transepidermal water loss (TEWL) on lateral upper arm and volar forearm in 198 infants, recruited from the general population in Norway, with no eczema, possible atopic aczema (AE) and AE. Measurements were performed three times and recorded as the estimated mean g/m^2^h-1 with (95% CI) at each site analysed by robust regression analysis.
(DOCX)

S1 Text Environmental impact on TEWL. In the post-hoc analysis performed to assess the potential impact on TEWL by room air humidity and temperature in all but seven crying infants (n = 233), room air temperature, but not humidity was correlated with TEWL (see main manuscript). Details of the extended study population is given in S1 Table, demonstrating that the 198 included infants were similar to those 42 infants who were remained excluded due to ambient conditions or crying.
(DOCX)
