# Supplementary material for: Skin Barrier Function and Staphylococcus aureus Colonization in Vestibulum Nasi and Fauces in Healthy Infants and Infants with Eczema: A Population-Based Cohort Study
Source: PLoS One. 2015 Jun 12;10(6):e0130145. doi: 10.1371/journal.pone.0130145 (PMC4466520; doi:10.1371/journal.pone.0130145)
Supplement: S1 Table — Characteristics of 198 included and 42 excluded infants enrolled as a control population for the Bronchiolitis All SE-Norway study. Infants were excluded due to crying (n = 7) and/or not fulfilling strict environmental criteria for humidity and/or temperature (n = 35). All values are given as number (percentage), unless otherwise stated. (DOCX) [file pone.0130145.s001.docx]

**S1 Table.**

|  | **Included infants**  **n=198** | **Excluded infants**  **n=42** | **p-values** |
| --- | --- | --- | --- |
| **Male** | 105 (53) | 28 (68) | 0.07 |
| **Age (months (min-max))** | 6.5 (1.0-13.4) | 6.8 (1.2-13.6) | 0.72 |
| **No eczema** | 129 (65) | 29 (71) | 0.27 |
| **Possible atopic eczema** | 34 (17) | 3 (7) | 0.27 |
| **Atopic eczema** | 35 (18) | 9 (22) | 0.27 |
| **Mother Caucasian** | 185 (93) | 40 (98) | 0.84 |
| **Father Caucasian** | 185 (93) | 36(88) | 0.31 |
| **Smoking at home** | 7 (4) | 3 (7) | 0.29 |
| **Cat at home** | 21(11) | 7(17) | 0.28 |
| **Dog at home** | 22 (12) | 3 (7) | 0.43 |
| **Parental eczema** | 66 (34) | 10 (24) | 0.26 |
| **Parental asthma** | 55 (28) | 9 (22) | 0.44 |
| **Parental rhinitis** | 106 (54) | 25 (61) | 0.38 |
| ***Filaggrin* mutation** | 15 (8) | 1 (2) | 0.22 |
| ***S. aureus* vestibulum nasi** | 53 (27) | 7 (16) | 0.17 |
| ***S. aureus* fauces** | 85 (43) | 16 (38) | 0.56 |
| ***S.aureus* vestibulum nasi/fauces** | 104 (53) | 18 (43) | 0.23 |
