# Supplementary material for: Skin Barrier Function and Staphylococcus aureus Colonization in Vestibulum Nasi and Fauces in Healthy Infants and Infants with Eczema: A Population-Based Cohort Study
Source: PLoS One. 2015 Jun 12;10(6):e0130145. doi: 10.1371/journal.pone.0130145 (PMC4466520; doi:10.1371/journal.pone.0130145)
Supplement: S2 Table — Transepidermal water loss (TEWL) on lateral upper arm and volar forearm in 198 infants, recruited from the general population in Norway, with no eczema, possible atopic aczema (AE) and AE. Measurements were performed three times and recorded as the estimated mean g/m2h-1 with (95% CI) at each site analysed by robust regression analysis. (DOCX) [file pone.0130145.s002.docx]

**S2 Table.**

|  | **No eczema** | **Possible atopic eczema** | **Atopic eczema** | **Overall p-value** |
| --- | --- | --- | --- | --- |
|  | **n=129** | **n=34** | **n=34** |  |
| **Lateral upper arm** | 7.9 (7.2-8.6) | 8.4 (6.9-9.8) | 10.8 ^a^(9.4, 12.3) | 0.002 |
| **Volar forearm** | 9.6 (8.6-10.6) | 9.8 (7.9-11.7) | 12.3^b^ (10.4-14.2) | 0.04 |

^a^ p=0.0004 and ^b^ p=0.01 atopic eczema vs. possible atopic eczema. There was no significant difference between possible atopic eczema and no eczema.
