# Supplementary material for: Skin Barrier Function and Staphylococcus aureus Colonization in Vestibulum Nasi and Fauces in Healthy Infants and Infants with Eczema: A Population-Based Cohort Study
Source: PLoS One. 2015 Jun 12;10(6):e0130145. doi: 10.1371/journal.pone.0130145 (PMC4466520; doi:10.1371/journal.pone.0130145)
Supplement: S1 Text — In the post-hoc analysis performed to assess the potential impact on TEWL by room air humidity and temperature in all but seven crying infants (n = 233), room air temperature, but not humidity was correlated with TEWL (see main manuscript). Details of the extended study population is given in S1 Table, demonstrating that the 198 included infants were similar to those 42 infants who were remained excluded due to ambient conditions or crying. (DOCX) [file pone.0130145.s003.docx]

**S1 Text. Environmental impact on TEWL.** In the post-hoc analysis performed to assess the potential impact on TEWL by room air humidity and temperature in all but seven crying infants (n= 233), room air temperature, but not humidity was correlated with TEWL (see main manuscript). Details of the extended study population is given in supplementary Table 1, demonstrating that the 198 included infants were similar to those 42 infants who were remained excluded due to ambient conditions or crying.
